# HDAC8-dependent deacetylation of PKM2 directs nuclear localization and glycolysis to promote proliferation in hepatocellular carcinoma

**DOI:** 10.1038/s41419-020-03212-3

**Published:** 2020-12-05

**Authors:** Ruixue Zhang, Mengqin Shen, Chunhua Wu, Yumei Chen, Jiani Lu, Jiajin Li, Li Zhao, Huannan Meng, Xiang Zhou, Gang Huang, Xiaoping Zhao, Jianjun Liu

**Affiliations:** 1 Department of Nuclear Medicine, Renji Hospital, School of Medicine, Shanghai Jiao Tong University, Shanghai, 200127 China; 2 Division of Physical Therapy Education, University of Nebraska Medical Center, Omaha, NE USA; 3 Shanghai University of Traditional Chinese Medicine, Shanghai, 201203 China; 4 Shanghai Key Laboratory of Molecular Imaging, Shanghai University of Medicine and Health Sciences, Shanghai, 201318 China

**Keywords:** Cancer metabolism, Gastrointestinal cancer

## Abstract

Pyruvate kinase M2 (PKM2) is not only a key rate-limiting enzyme that guides glycolysis, but also acts as a non-metabolic protein in regulating gene transcription. In recent years, a series of studies have confirmed that post-translational modification has become an important mechanism for regulating the function of PKM2, which in turn affects tumorigenesis. In this study, we found that K62 residues were deacetylated, which is related to the prognosis of HCC. Further studies indicate that HDAC8 binds and deacetylates the K62 residue of PKM2. Mechanistically, K62 deacetylation facilitate PKM2 transport into the nucleus and bind β-catenin, thereby promoting CCND1 gene transcription and cell cycle progression. In addition, the deacetylation of K62 affects the enzyme activity of PKM2 and the flux of glucose metabolism. Therefore, these results suggest that HDAC8 / PKM2 signaling may become a new target for the treatment of HCC.

## Introduction

Liver cancer is predicted to be the sixth most commonly diagnosed cancer and the fourth leading cause of cancer death worldwide in 2018, and its ranks fifth in terms of global cases and second in terms of deaths for males, and hepatocellular carcinoma (HCC) is the most prevalent form one^1^. New pathophysiology directed therapies are urgently needed. Therefore, with extensive studies, an increasing number of molecular mechanisms of pathogenesis have been discovered^2–4^.

PKM2 is overexpressed in HCC, and participates in hepatocarcinogenesis and progression through many critical signaling networks, including PRMT6-ERK-PKM2 regulatory axis, circMAT2B/miR-338-3p/PKM2 axis and others^5–7^. It not only acts as a key enzyme in anaerobic glycolysis in the cytoplasm, but also play a major role in functions as a histone kinase in the nucleus^8–10^. Further studies have shown these alterations are regulated by various PTMs, including phosphorylation, acetylation, and so on^11–15^. Aberrant acetylation levels of PKM2 has been linked to the development of several diseases. For example, acetylation of K433 of PKM2 can bind to c-Src-phosphorylated Y333 of β-catenin. Moreover, this interaction is required for both proteins to be recruited to the *CCND1* promoter for promoting GBM progression^8^. The mentioned above site and K305 residues of PKM2 also could be deacetylated by SIRT2 and SIRT 6 respectively, both of kinase and enzyme functions were weakened^16,17^. Here we try to find new functional site and further characterize PKM2 function in HCC.

Histone deacetylase 8 (HDAC8), one of the crucial HDACs, plays an important role in occurrence and progression of various diseases, including cancer, hereditary disease, and parasitic infections via different signaling pathways^18,19^. It can drive NAFLD-associated hepatocarcinogenesis through inhibiting p53/p21-mediated apoptosis and cell-cycle arrest and stimulating β-catenin-dependent cell proliferation^3^. While up to half of HCC patients have activation of Wnt/β-catenin signaling pathway^8,20^. This signaling pathway plays a significant role in the physiology and pathology of liver and has been a prevalent therapy target^21^.

Both of PKM2 and HDAC8 proteins are overexpressed in tumor tissues. They can impact cell cycle by influencing β-catenin in hepatocellular carcinoma (HCC). Previous study revealed PKM2 binds with several proteins that regulate glucose and lipid metabolism then promote cell proliferation in HCC^22^. In this study, we found that HDAC8 directly interacts with PKM2 and deacetylates the conserved K62 residue in the cytoplasm, thereby regulating glucose metabolism and gene transcription. Clinically, the acetylation level of PKM2-K62 residues is significantly correlated with the prognosis of HCC. This study reveals a novel regulatory mechanism of PKM2 protein and suggests its potential significance in HCC treatment.

## Materials and methods

### Patient tissue specimens

HCC specimens and adjacent tissues obtained from the surgical specimen archives of Renji Hospital, School of Medicine, Shanghai Jiaotong University. This research was approved by the institutional clinical research ethics committee of Renji Hospital, School of Medicine, Shanghai Jiaotong University. None of these patients had received radiotherapy or chemotherapy prior to surgery. And, before surgery, all of them received ^18^F-FDG PET/CT examination. The diagnosis of all HCC cases was validated by pathological examinations.

### Cell lines and reagents

HEK293T, SMMC-7721, HepG2 were obtained from ATCC (Manassas, VA, USA); LO2 was obtained from the Cell Research Institute of the Chinese Academy of Sciences (Shanghai, China); they were cultured in DMEM (GIBCO, Grand Island, NY, USA) supplemented with 10% FBS (GIBCO). The antibodies used were anti-Flag (20543-1-AP, Proteintech), anti-HA (901533, Biolegend), anti-PKM2 (3198S Cell Signaling Technology), anti-HDAC8 (ab187139, Abcam), anti-Cyclin D1 (ab16663, Abcam), β-catenin(8480S, Cell Signaling Technology), Non-phospho (Active) β-Catenin (Ser45) (19807, Cell Signaling Technology), PCI-34051(S2012, Selleck, Houston, USA). TM-2-51(S505528-1G, Sigma-Aldrich, USA).

### Co-immunoprecipitation assay

To analyze exogenous protein–protein interaction, 10 μl of anti-HA beads (Sigma-Aldrich, USA) was incubated with the cell lysate that included HA or Flag-tagged PKM2 and HDAC8 protein overnight at 4 °C. For endogenous Co-IP assay, cell lysate was incubated with antibody against PKM2 and 10 μl protein A/G agarose (Pierce, Dallas, USA) overnight at 4 °C. The precipitates were washed four times with lysis buffer, and then, suspended in 5× SDS-PAGE sample loading buffer. After boiling for 5 min, the samples were analyzed by western blotting and detected by the relevant antibodies.

### GST pull-down assay

BL21 (DE3, TansGen Biotech, Beijing, China) were used to express GST fusion PKM2 and His fusion HDAC8. The fusion proteins were purified with Glutathione-Sepharose 4B beads (GE Healthcare, Boston, USA) or Ni affinity resins (GE Healthcare) according to the manufacturer’s instructions. The GST fusion proteins were immobilized on glutathione-sepharose beads and then incubated with His-HDAC8 effluent at 4 °C for night. The beads were pelleted, washed by PBST (0.5% Tween-20 in PBS) for 5 times. Samples were analyzed by SAS-PAGE and Coomassie Blue Staining.

### Confocal immunofluorescence microscopy and proximity ligation assay (PLA)

SMMC-7721 and LO2 were grown in 24-well plates on glass coverslips. Cell fixation was performed using 4% paraformaldehyde in PBS. After permeabilization with 0.25% Triton X-100, the cells were treated with blocking buffer for 30 min and incubated overnight at 4 °C with the primary antibody, followed by incubation with the secondary antibody at room temperature (RT) for 1 h. The coverslips were mounted onto glass slides and counterstained with DAPI. Confocal laser-scanning microscope (Olympus BX61) was used to observe the image (Sigma-Aldrich, USA).

PLA was performed according to the manufacturer’s protocol (Duolink in situ PLA, Sigma-Aldrich). Before the experiment, the cells were processed as confocal immunofluorescence microscopy, and the end of the experiment, images were obtained as above.

### Mass spectrometry for acetylation modification

HepG2 and the corresponding HDAC8 knocked-out cells were overexpressed Flag-tagged PKM2 plasmid. After 48 h, cell lysates were treated as previously mentioned^23^.

### RNA interference and re‐expressed cell lines

Lipofectamine 2000 (Invitrogen, Waltham, MA, USA) was used in transient transfection according to the manufacturer’s protocol. The sequences of siRNA oligos were as follows: PKM2 (sense 5ʹ GCCCGAGGCTTCTTCAAGAAGTT-3ʹ, antisense 5ʹ-CTTCTTGAAGAAGCCTCGGGCTT-3ʹ), HDAC8 (sense 5ʹ-GCAGAUGAGGAUAGUUAAG TT-3ʹ, antisense 5ʹ-CUUAACUAUCCUCAUCUGC TT-3ʹ) and negative control: (sense 5ʹ-UUCUCCGAACGUGUCACGUTT-3ʹ, antisense 5ʹ-ACGUGACACGUUCGGAGAATT-3ʹ).

For PKM2‐re‐expressed cell lines, cells were infected by lentivirus with shPKM2 (sense 5ʹ‐CCGGGCCCGAGGCTTCTTCAAGAAGCTCGAGCTTCTTGAAGAAGCCTCGGGCTTTTTG‐3ʹ, antisense 5ʹ‐AATTCAAAAAGCCCGAGGCTTCTTCAAGAAGCTCGAGCTTCTTGAAGAA GCCTCGGGC‐3ʹ) first. Single colonies were isolated by limited dilution and expansion. Clones were then genotyped by sequencing and validated by immunoblotting. Following by infected by pLenti‐CMV‐Cherry‐3Flag‐PGK‐Puro- PKM2 virus (WT/K62R/K62Q).

### RNA extraction and real-time PCR assay

Total RNA was isolated from the cultured cells with TRIzol Kit (Omega, Norcross, GA, USA). Reverse transcription was performed using the cDNA synthesis kit (Takara, Otsu, Japan) following the manufacturer’s instructions. Real-time PCR was carried out by using SYBR green fluorescence (Takara). The StepOnePlus Real-Time PCR System (Thermo Fisher Scientific) was used to carry out the quantitative PCR. The primers for Cyclin D1were as follow: 5ʹ- GCTGCGAAGTGGAAACCATC-3ʹ (sense) and 5ʹ- CCTCCTTCTGCACACATTTGAA - 3ʹ (antisense). PKM2 5ʹ- GGGCCATAATCGTCCTCACC -3ʹ (sense) and 5ʹ-TTGCACAGCACAGGGAAGAT-3ʹ (antisense). HDAC8 5ʹ-TCGCTGGTCCCGGTTTATATC-3ʹ (sense) and 5ʹ-TACTGGCCCGTTTGGGGAT-3ʹ (antisense)

### Cell proliferation assay

For colony formation assay, Cells were put into 6-well plates at a density of 1 × 10^3^ per well, stained with Crystal violet after 10 days. For CCK8, according to the instructions of Cell Counting Kit-8 (CCK-8, Bimake, Shanghai, China), 1 × 10^4^ Cells were put in 96-well per well, counting at 450 nm for four consecutive days.

5-Ethynyl-2′-deoxyuridine (EdU) detection kit (RiboBio, Guangzhou, China) experiment was carried according to the manufacturer’s instruction. Cells were putting into 96-well by 1 × 10^4^ per well after transfected by corresponding plasmids 24 h. Another 24 h passed, we fixed the cells by 4% paraformaldehyde in PBS, then other steps according to the manufactures’ instructions. Images were obtained by fluorescence microscopy (Olympus, Tokyo, Japan).

### PK activity, lactate, GSH, and NADP(H) assay

Cells were plated in 6-well plate. After different treatment, cell supernatant was collected to measure lactate concentration, while the cell pellets to be lysed and measured PKM2 activity, GSH, and NADP(H). All the experiments were conducted according to (Nanjing Jiancheng Bioengineering Institute, Nanjing, China).

### Extracellular acidification rate (ECAR) assays

The extracellular acidification rate (ECAR) were measured using Seahorse XF Glycolysis Stress Test kit (Agilent Technologies). The Seahorse XF 24 Extracellular Flux Analyzer (Seahorse Bioscience) was used according to a previously described method^24^.

### ^14^CO_2_ release assay

The incorporation of [1-^14^C] glucose or [6-^14^C] glucose into ^14^CO_2_ was determined as previously reported^25^. Briefly, cells were cultured in 10 cm^2^ dishes, and the cells were exposed to DMEM supplemented with [1-^14^C] glucose (0.1 µCi/ml) or [6-^14^C] glucose (0.1 µCi/ml). The dish was placed in a container to collect CO_2_ produced. Rates of glucose consumption were measured by incubating cells for 120 min at 37 °C. Fresh air was pumped into the container by a ventilator. The [^14^C] CO_2_ was driven into a vial and trapped by Hyamine hydroxide. No-cell controls were included to correct for unspecific CO_2_ trapping.

### Mass spectrometry analysis

When Density was 90% per 10 cm dish, Cells were blowed down gently and washed by NS quickly, then transferring to 1.5 ml tube (1/10 was reserved for protein quantification). Samples were extracted by cell metabolite extract (ACN/MeOH/Water: 40/40/20, −20 °C for pre-cooling one night). The cell pellets were put on ice, 300 µl extract buffer was added per tube. The cell precipitation was thoroughly blown and mixed, then it was swirled on the vortex instrument for 10 s and then put into the refrigerator at minus 20 degrees Celsius for 20 min. Finally, supernatant was obtained by centrifugation (12,000 × *g*, 4 °C for 10 min). In case of precipitation, only 200 µl volume of supernatant was transferred. The samples were tested on LC-MS machine (Agilent Technologies, Calif., USA).

### Xenograft tumor studies

4-week male NCG mice were purchased from Renji Hospital Experimental Animal Center (Shanghai, China). All animal experiments were reviewed and approved by Renji Institutional Animal Care and Use Committee (Shanghai, China). Briefly, NCG mice were randomly divided into two groups (*n* = 4 per group) and inoculated subcutaneously with 1 × 10^7^ HepG2 cells in the right while with 1 × 10^7^ HDAC8-KO cells in the left flank. Similarly, HDAC8-KO/PKM2-WT cells and HDAC8-KO/PKM2-K62R cells were injected subcutaneously into the right and left flank of mice with 1 × 10^7^ cells per injection, respectively. The volumes of tumors were measured as the length × width^2^ × 0.5. After 5 weeks, PET imaging scans were carried out on a micro-PET/CT scanner (Super Nova®PET/CT, PINGSENG Healthcare Inc., Shanghai, China). 18F-FDG (~200 µl, ~7.4 MBq) was injected into the tail vein of tumor-bearing mice. After 30 min, the animals were anesthetized with 2% isoflurane and immobilized during PET scan acquisition. Maximum standard uptake value (SUVmax) was used to assess the ^18^F-FDG uptake by tumors. After imaging, mice were sacrificed and tumors were excised.

### Statistical analysis

The data analysis was performed by using statistical program SPSS19.0 (IBM, USA) and Graph Pad Prism 7.0 (Graph-Pad Software, Inc., USA). The quantitative data were expressed as mean ± SEM. The variance was similar between the groups that are being statistically compared. No samples or mice were excluded from the analysis as outliers. Student’s *t* test, Chi-square test and one way ANOVA was used to determine differences between groups upon the data type. Kaplan–Meier method was used to calculate survival curves. *p* values < 0.05 were regarded as statistically significant.

## Results

### Acetylation of PKM2 lysine residue 62 impacts prognosis of HCC

**Fig. 1:**
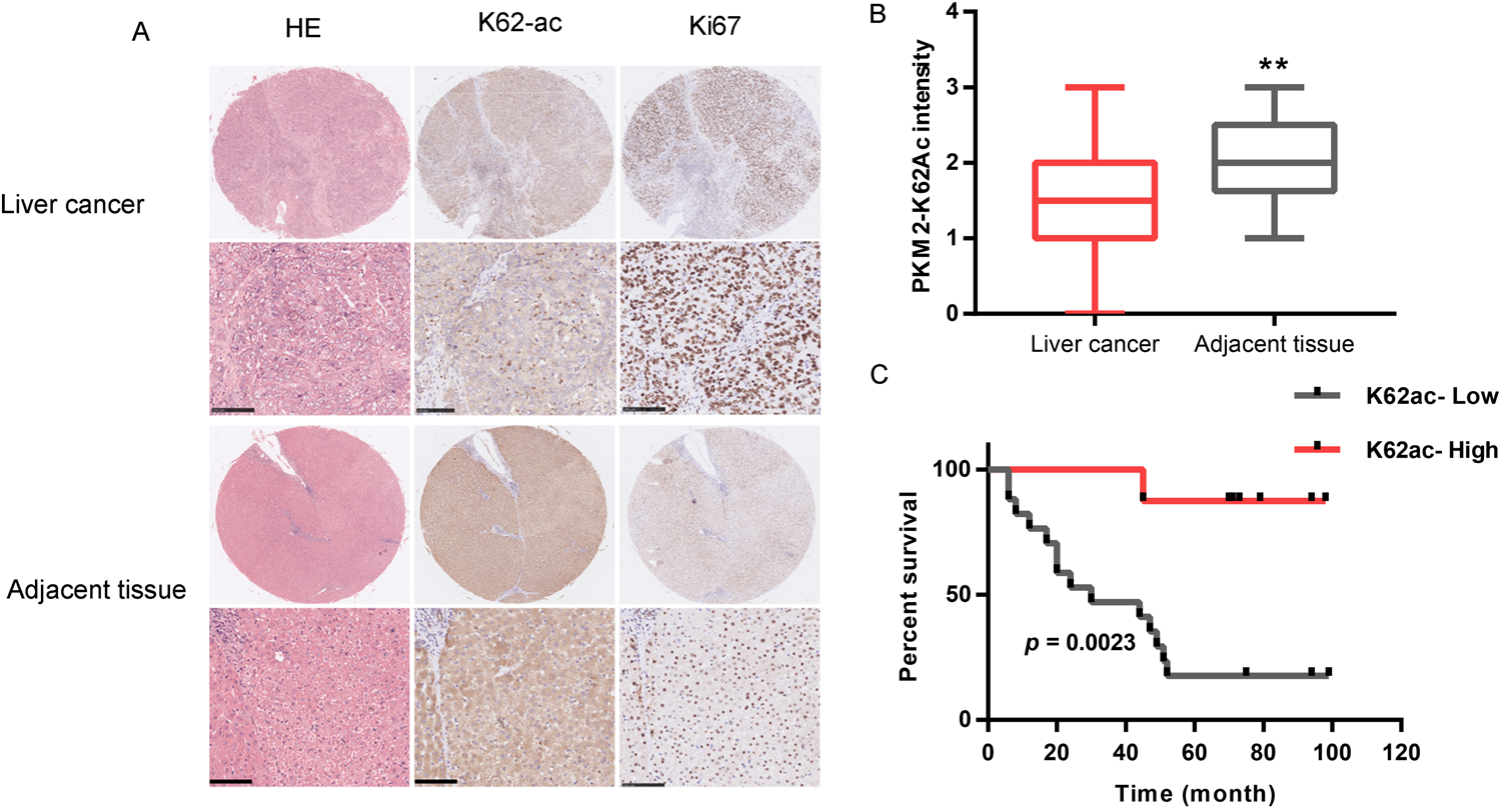
Deacetylation of K62 promotes tumor growth of hepatocellular carcinoma. **A** HCC and its adjacent normal tissues were typically histopathologically analyzed using H&E staining and ki67, PKM2-K62-ac antibodies (n = 31). **B** Comparison of immunohistochemical staining scores of PKM2-K62-ac between cancer and adjacent tissues (**p < 0.01); **C** Overall survival analysis by Kaplan–Meier (low and high staining groups were categorized according to the immunohistochemistry scores and Scale bar, 100 µm).

PKM2 plays an important role in tumor development and progression through the regulation of both metabolic and nonmetabolic pathways^26^. To evaluate the expression differences of PKM2 between tumors and normal tissues in HCC, we applied the gene expression profiling interactive analysis (GEPIA) to analyze the data from TCGA and GTEx projects. PKM2 expression was higher in HCC compared with adjacent normal samples (Fig. S1A). We also further used GEPIA to analyze the prognostic value of PKM2 in HCC. Results revealed that high PKM2 expression levels were associated with poorer prognosis of OS in HCC (OS Log-rank *p* = 0.005, HR = 1.8) (Fig. S1B). In GSE4465, genomic profiling is performed in HepG2 cells after TSA (0.5 µM) treatment. As shown in Fig. S1C, PKM2 levels are upregulated upon HDAC inhibition by TSA treatment. There is clear evidence that the acetylation of PKM2 lysine residues 305 (K305) and 433 (K433) regulates metabolic flux and gene transcription, ultimately affecting the malignant phenotype of the tumor^16,17,27–29^. There are 24 potential lysine sites in PKM2 that can be modified by acetylation (Fig. S1D, E), but their role in tumors is unclear. Lv, L et al. demonstrate that PKM2 Lysine residue 62 (K62) is acetylated, but not the target of SIRT2^27^. Therefore, there is great interest to test role of K62 acetylation of PKM2 in tumor development and progression. To gain insight of post-translational regulation of PKM2 at K62 residue, we generated an antibody that specifically recognizes K62 acetylation (Fig. S1F, G) to determine its clinical significance in HCC tissues. We evaluated the K62 acetylation level of PKM2 in paired tumors and adjacent normal tissues in 31 HCC patients. The K62 acetylation level of PKM2 in the tumor is lower than that of adjacent tissues, and it is accompanied by rapid cell proliferation (Fig. 1A). Statistical analysis shows that K62 acetylation level of PKM2 is significantly lower than that of adjacent tissues (Fig. 1B). As shown in Table 1, K62 deacetylation level of PKM2 was significantly correlated with tumor size (*p* = 0.041), sample grade (*p* = 0.046), glucose uptake (SUVmax, *p* = 0.036) and hepatocirrhosis (*p* < 0.019), but not with age, portal vein invasion, or HBV infection (Table 1). More importantly, PKM2 K62 deacetylation level is closely related to the prognosis of HCC patients (5-year survival rate, *p* < 0.001). The survival time of patients with low K62 acetylation was significantly shorter than that of patients with high K62 acetylation (*p* < 0.001) (Fig. 1C). Taken together, these data indicate that K62 deacetylation of PKM2 impacts prognosis of HCC, and K62 deacetylation may play an important role in the development and progression of HCC.

**Table 1:** Relationship between acetylation of K62and clinicopathological parameters of hepatocellular carcinoma patients.

PKM2-K62Ac				
Characteristics	All cases	High	Low	*P* value
Participaints	31	14	17	
Age				0.578
< 50 years	15	7	8	
≥ 50 years	16	7	9	
Tumor size				0.041
< 5 cm	16	10	6	
≥5 cm	15	4	11	
Hepatocirrhosis				0.019
Negative	7	6	1	
Positive	24	8	16	
Sample grade				0.046
1	5	4	1	
2	20	9	10	
3	6	1	6	
5-Year survival rate				< 0.001
<5 years	14	1	13	
≥5 years	11	10	1	
SUVmax				0.036
<5.3	18	11	7	
≥5.3	10	2	8	
Portal vein invasion				0.195
Negative	23	12	11	
Positive	7	2	5	
HBV infection				0.394
Negative	13	5	8	
Positive	18	9	9	

### HDAC8 regulates K62 acetylation of PKM2

**Fig. 2:**
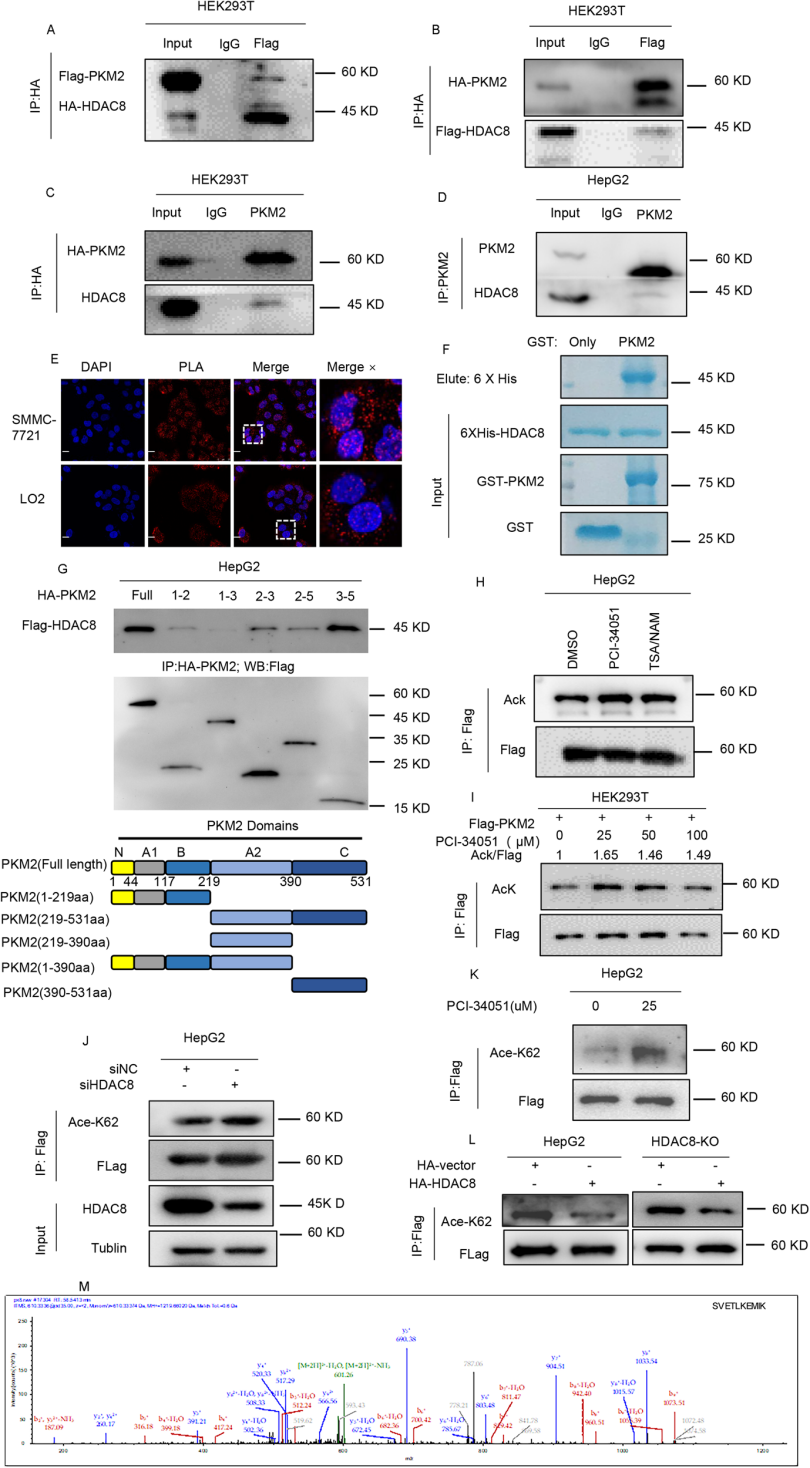
HDAC8 deacetylates PKM2 at lysine-62. HEK293T cells were transfected with Flag- PKM2 / HA-HDAC8 (**A**) or Flag- HDAC8/HA- PKM2 (**B**). After 48 h, HA-tagged protein was immunoprecipitated from HEK293T cells lysates. Then, immunoprecipitates and whole-cell extracts were subsequently separated by SDS-PAGE and immunoblotted with Flag and HA antibodies. **C** HepG2 cells were transfected with HA- PKM2 for 48 h, Extracts were immunoprecipitated with anti-HA (IgG as control). Immunoprecipitates and whole-cell extracts were subsequently analyzed by western blotting with an anti-HDAC8 and HA antibody. **D** Lysates of HepG2 cells were immunoprecipitated with the antibody against PKM2. Immunoprecipitates and whole-cell extracts were analyzed by western blotting with anti-PKM2 and anti-HDAC8 antibodies. **E** The Duollink PLA technology showed the interaction(red) between HDAC8 and PKM2 in SMMC-7721 and LO2 cells (Scale bar, 50 µm). **F** The mixture of recombinant GST- PKM2(GST-vector as a control) and His- HDAC8 were rotated and incubated for 3 h. Bound proteins were eluted, then analyzed with Coomassie Blue staining. **G** FLAG-HDAC8 was co-expressed in HEK293T cells with HA-PKM2 fragments as indicated. Co-IP followed by western blotting was performed to determine their interaction. **H**, **I** After transfected with Flag- PKM2 24 h, PCI-34051 (25 µM) and TSA/NAM (1 µM /10 mM) (**H**) or different doses of PCI-34051 (**I**) are added for 24 h. Cell lysates were immunoprecipitated with Flag affinity gel. PKM2 acetylation and protein levels were analyzed by immunoblotting using the indicated antibodies. **J–L** Interfered by siHDAC8 (**J**), PCI-34051 (**K**), and HA-HDAC8 (**L**) in HepG2 cells or HDAC8-KO HepG2 cells, separately. After immunoprecipitated with Flag affinity gel, acetylation of K62 was determined by western blotting. **M** LC-MS-MS of PKM2 showing the acetylation of PKM2 K62 in HDAC8-KO HepG2 cells. HepG2 cells and HDAC8-KO HepG2 cells transfected with Flag-PKM2. The immunoprecipitates were sent for LC-MS-MS analysis of the acetylation intensity of PKM2 peptides.

Next, we explored who is responsible for regulating the acetylation level of PKM2-K62 residue. Given that deacetylase or acetylase regulates the level of acetylation after binding to PKM2, we analyzed potential PKM2 binding partners by mass spectrometry and revealed a novel protein interaction between PKM2 and HDAC8 (data not shown). Several studies have shown that HDAC8 deacetylates non-histone proteins, thereby affecting the growth of tumor cells^30–32^. Therefore, we hypothesize that HDAC8 may be a potential deacetylase of PKM2 protein. Through a series of exogenous or endogenous protein interaction analysis, we found that HDAC8 and PKM2 had protein–protein interaction (Fig. 2A–D). And this interaction localized in both cytoplasm and nuclear of SMMC-7721 and LO2 cells (Fig. 2E). In vitro binding experiments revealed that HDAC8 could directly interact with PKM2 (Fig. 2F). Based on the characteristics of the PKM2 protein domain, we found that the 390–531aa is the key sequence of PKM2 binding to HDAC8 (Fig. 2G). These protein–protein interaction results strongly suggest that HDAC8 may be a regulator of PKM2 protein. Compared with the control group, the overall acetylation level of PKM2 increased after treatment with HDAC8 specific inhibitor PCI-34051or TSA/NAM (Fig. 2H), which is consistent with the previous reports^16,27,28^.

In the meantime, with the increase of HDAC8 inhibitor concentration, the overall level of PKM2 acetylation also moderately upregulated (Fig. 2I). More importantly, knocked down HDAC8 or inhibition its activity significantly increased the level of acetylation at the K62 site of the PKM2 protein, which was more pronounced than the overall level of acetylation (Fig. 2J, K). To further analyze the specificity of HDAC8 on the regulation of PKM2 acetylation, we established HDAC8 knockout HepG2 cells (Fig. S2A). Then, we analyzed the change of K62 acetylation in the absence or presence of HDAC8. As shown in Fig. 2L, the loss of HDAC8 lead to an increase in K62 acetylation levels, which is consistent with the effect of HDAC8 inhibitor. However, in the case of HDAC8 overexpression, the acetylation level of K62 decreased accordingly (Fig. 2L).

Then, we verified the effect of HDAC8 on the acetylation of PKM2 at the K62 residue by mass spectrometry (Fig. 2M and S2B). Therefore, these results indicate that HDAC8 can bind PKM2 and deacetylate its K62 residue.

### Deacetylation of K62 reprograms glucose metabolism

**Fig. 3:**
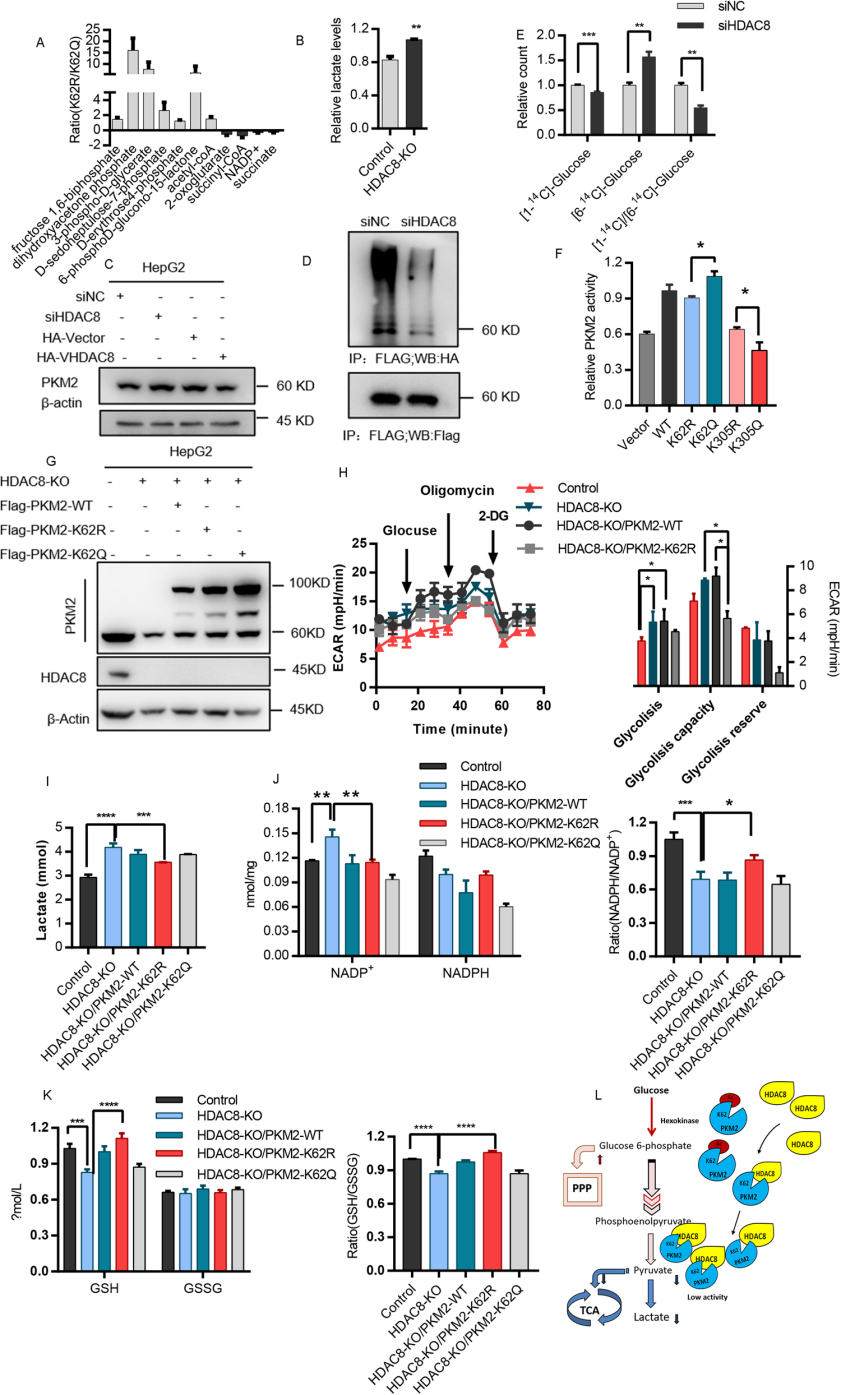
Deacetylation of K62 reprograms glucose metabolism. **A** Mutants at K62 (R/Q) of PKM2 were transferred into HepG2 cells, after 48 h, cells were collected and extracted for LC-MS. **B** Medium supernatant of HepG2 and HDAC8-KO HepG2 cells in 6-well plate were collected and measured lactate production, protein quantification for calibration. **C** HepG2 cells expressing si-HDAC8 (si-NC) or HA-HDAC8 (HA-vector) plasmids. After 48 h, cell lysates were analyzed by western blotting with indicated antibodies. **D** HepG2 cells were transfected with siHDAC8(siNC as a control), after 6 h, followed by co-transfected with Flag-PKM2 and HA-UB plasmid, 42 h after transfection, cells were incubated with MG132 (100 μM) for 6 h. Cell lysates were immunoprecipitated with anti-flag antibody followed by immunoblotting analysis with anti-HA or anti-flag antibody. **E** After interfering with HDAC8 expression level by siHDAC8 for 48 h, the incorporation of [1-^14^C] glucose or [6-^14^C] glucose into ^14^CO_2_ was determined. **G** The construction of cell lines which were stably expressing PKM2 plasmids (WT/K62R/K62Q) in HDAC8-KO HepG2 cell lines. **H** 15,000 cells per well were planted in Seahorse XF plate, cultured overnight, and then texted ECAR according to protocol. **I**–**K** A mass of above-mentioned cells (**G**) were collected and treatment by the corresponding lysis buffer, and measured lactate (**I**) NADP^+^/NADPH (**J**) and GSH/GSSG (**K**) according to protocols. **l** A summary of glucose metabolism pathway regulated by HDAC8 in HCC. (Data are mean ± SEM, *n* = 3–5, *p < 0.05, **p < 0.01, ***p < 0.001. Student’s *t* test in (**A**, **B**, **E**), one way ANOVA in (**H**, **I**, **J**, **K**).

PKM2 catalyzes the final and rate-limiting reactions in the glycolysis pathway and plays an important role in glucose metabolism. Through metabolic mass spectrometry, we found that the acetylation status of K62 affects the metabolic phenotype of tumor cells (Fig. 3A). HDAC8 not only regulated the enzyme activity of PKM2 (Fig. 3B), but also affected the protein stability of PKM2 (Fig. 3C). Knockdown of HDAC8 reduced the ubiquitination level of PKM2 (Fig. 3D), resulting in a slight accumulation of PKM2 protein (Fig. 3C). Since HDAC8 was found to be a new upstream regulator of PKM2, then we analyzed the effect of HDAC8 on glucose metabolism of HCC cells. The C1 of glucose mainly flux to PPP and TCA, while the C6 mainly flux to TCA for metabolism. Through [1-^14^C] glucose or [6–^14^C] glucose tracer analysis, we found that after HDAC8 knockdown, the glucose metabolism through the PPP pathway decreased, while the metabolism through the TCA pathway increased accordingly (Fig. 3E). HDAC8 can regulate glucose metabolism through enzyme activity dependent or independent manners. As shown in Fig. 3F, the enzyme activity of PKM2 of K62R is significantly lower than that of K62Q. In order to exclude endogenous interference, we expressed PKM2 wild-type, PKM2-K62R or PKM2-K62Q mutant in HDAC8 knockout cells to analyze its impact on cancer cell metabolism (Fig. 3G). The PKM2-K62R mutant was able to partially compromise increase of glycolysis (Fig. 3H) and lactate (Fig. 3I) levels that induced by HDAC8 knockout. It was further supported that by analyzing the levels of NADPH/NADP ^+^ and GSH/GSSG (Fig. 3J, K), we found that the PKM2-K62R mutant impaired the ability of cells to respond to oxidative stress after HDAC8 knockout. Therefore, these results indicate that HDAC8 reprograms the glucose metabolism of HCC cells by regulating K62 acetylation of PKM2 protein, so that glucose is mainly metabolized through the PPP pathway to cope with the challenge of oxidative stress (Fig. 3L).

### Deacetylation at K62 promotes nuclear translocation of PKM2

**Fig. 4:**
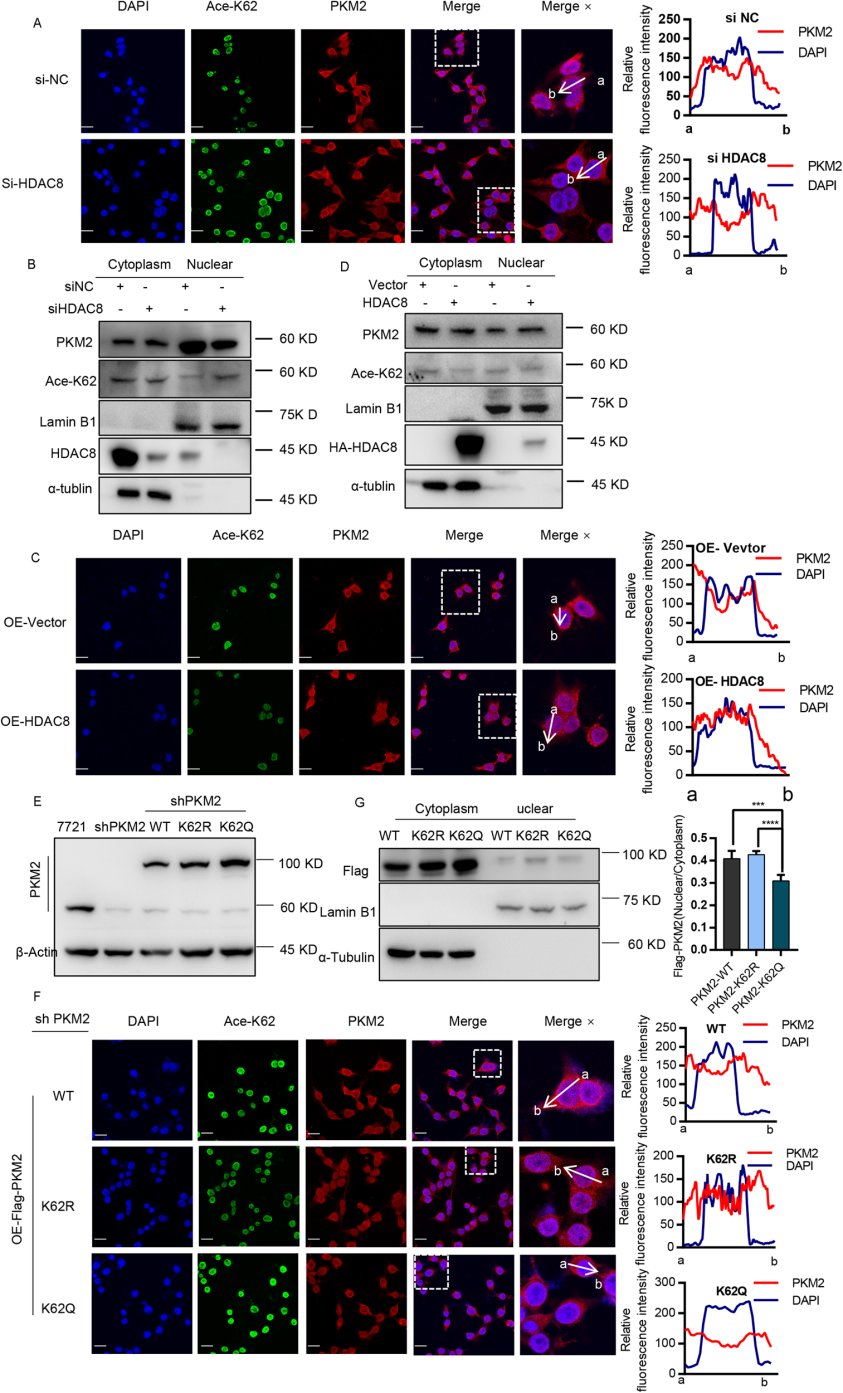
Deacetylation at K62 promotes nuclear translocation of PKM2. **A**–**D** HepG2 cells were transfected with siHDAC8 (or negative control siRNA) or HA-HDAC8 plasmids (or vector plasmid), after 24 h, cells were placed on glass coverslips, cultured for another 24 h. Anti-PKM2 (red) and anti-k62ac (green) were used for immunofluorescence (scale bar: 50 μm) (**A**, **C**). The line profiles of the mean fluorescence intensity of PKM2 and DAPI signals were measured by ImageJ software. The remaining cell pellets were separated by cytosolic and nuclear fractions (**B**, **D**) for western blotting. **E** The cell lines which were stably expressing Flag-mcherry-PKM2 plasmids (WT/K62R/K62Q) in shPKM2-HepG2 cell lines. Immunofluorescence technique was used like above description (**F**), the remaining cell pellets were separated by cytosolic and nuclear fractions (**G**) for western blotting (***p < 0.001, ****p < 0.0001, one way ANOVA).

Nuclear localized PKM2 plays an important role in regulating the growth of tumor cells through non-metabolic functions. Studies have demonstrated that EGF can stimulate PKM2 to translocate the nucleus, thereby binding and regulating the function of β-catenin^8,16^. Therefore, we analyzed the effect of EGF on the acetylation level of PKM2. As shown in Fig. S3, we observed that the K62 acetylation level of PKM2 was significantly reduced after EGF treatment. Furthermore, HDAC8 knockdown prevented the accumulation of PKM2 protein in the nucleus (Fig. 4A and S4A). By extracting nuclear proteins, we observed that similar phenomenon that HDAC8 regulated PKM2 nuclear localization (Fig. 4B). In contrast, HDAC8 overexpression was able to promote PKM2 translocation to the nucleus (Fig. 4C, D and S4B). In addition, both confocal fluorescence and nuclear protein analysis showed that the PKM2 mutant (K62R) lacking K62 acetylation had more nuclear enrichment than the acetylated PKM2 mutant (K62Q) (Fig. 4E–G). Therefore, these results indicate that HDAC8 promotes the entry of PKM2 into the nucleus by regulating the deacetylation of K62 residue.

### HDAC8 upregulates CCND1 expression and G1-S transition by promoting the binding of PKM2 to β-catenin

**Fig. 5:**
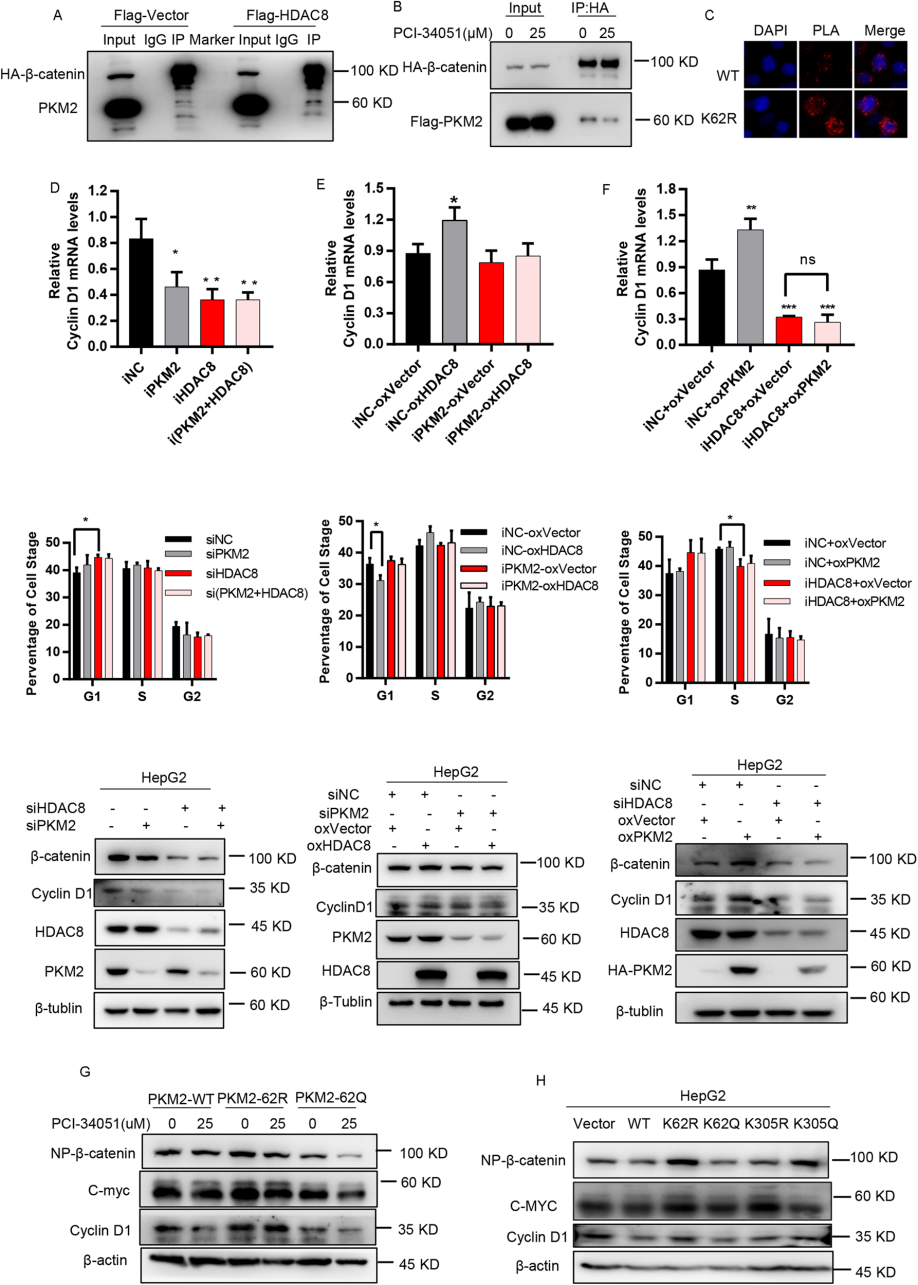
HDAC8-dependent deacetylation at K62 promotes the binding with β-catenin and upregulating cyclin D1 expression. **A** HepG2 cells were co-transfected with HA-β-catenin with Flag-HADC8 (Flag-vector), after 48 h, cells were collected for immunoprecipitated by HA beads, analyzed by western blotting with PKM2 and HA antibodies. **B** Co-transfected HA-β-catenin and Flag-PKM2 plasmids for 24 h, then PCI-34051 (25 µM, DMSO as control) added for another 24 h, immunoprecipitated by HA beads, western blotting using Flag and HA antibodies. **C** Cells were transfected by PKM2 WT or K62R plasmids, separately. After 48 h, the Duollink PLA technology was used to illustrate the interaction (red) of HA-PKM2and β-catenin in HepG2 cells (Scale bar, 50 µm). **D** HepG2 were transfected with siRNA against HDAC8 or PKM2 (negative control siRNA) for western blotting or qPCR. Another half were analyzed by cytometry. **E**, **F** Based on which siRNA were transfected, after 6 h, the cells were overexpressed another protein expression plasmid (controlled by negative siRNA and vector plasmid). Then experiments were carried out as mentioned above. **G** Cells were transfected PKM2 WT or mutant plasmids, separately. After 48 h, lysates of HepG2 were analyzed by western blotting using the indicated antibodies. **H** Based on above result. cells were transfected PKM2 wild type or K62 mutant plasmids, separately, then PCI-34051 (25 µM) was added after 24 h and maintained for 24 h. Cell lysates were analyzed by western blotting using the indicated antibodies. (one way ANOVA in (**D**–**F**), *p < 0.05, **p < 0.01).

PKM2-dependent β-catenin transactivation regulates CCND1 expression and cell cycle in cancer cells^8^. Therefore, we speculated whether HDAC8 can play a role in regulating the cell cycle through the PKM2/β-catenin complex. As shown in Fig. 5A, we observed that overexpression of HDAC8 promoted the binding of PKM2 to β-catenin. In contrast, HDAC8 inhibitor PCI-34051 inhibited the formation of PKM2/β-catenin complex (Fig. 5B). Compared with wild-type PKM2, the loss of acetylation at the K62 site promoted binding of PKM2 and β-catenin in the nucleus (Fig. 5C). Functionally, HDAC8 knockdown significantly reduced CCND1 expression and lead to G1 phase arrest, which was consistent with the phenomenon caused by PKM2 knockdown (Fig. 5D). In contrast, HDAC8 overexpression promoted CCND1 expression and cell cycle progression (Fig. 5E). However, HDAC8 overexpression and PKM2 knockdown did not result in changes in CCND1 expression and cell cycle (Fig. 5E). In addition, HDAC8 knockdown impaired PKM2-induced CCND1 expression and G1-S transition (Fig. 5F). Therefore, these results indicate that PKM2 as a downstream factor is necessary for HDAC8 to drive CCND1 expression and cell cycle progression.

To further elucidate the regulatory effect of K62 acetylation on the cell cycle, we expressed PKM2 wild-type and K62, K305 acetylation-related mutants in HepG2 cells. The result showed that K62 acetylation-deficient mutant (K62R) or K305 acetylation-mimic mutant (K305Q) promoted the expression of CCND1 (Fig. 5G), indicating that these two sites regulate CCND1 gene transcription through different mechanisms. In addition, in the presence of K62 acetylation (K62Q), rather than in the absence of K62 acetylation (K62R), a regulatory effect of HDAC inhibitor on CCDN1 expression was observed (Fig. 5H). In summary, these results indicate that HDAC8 promotes the expression of CCND1 and the transition of the cell cycle from G1 to S phase by deacetylating the K62 site of PKM2 protein.

### Deacetylation of PKM2 K62 residue promotes HCC cell growth

**Fig. 6:**
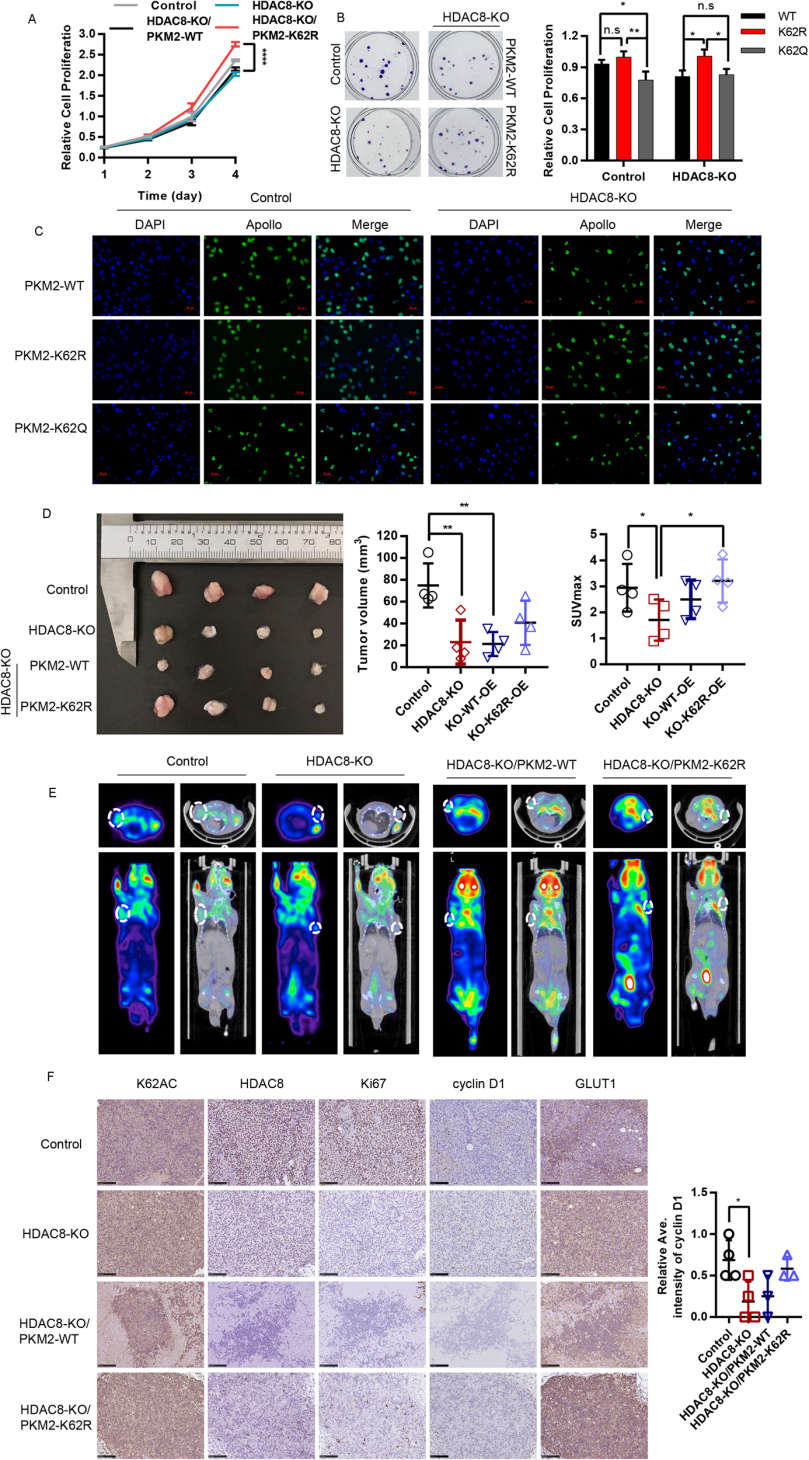
Deacetylation of K62 promotes cells proliferation in vivo and in vitro. CCK-8 assay (**A**), colony formation (**B**), and immunofluorescence analysis with EdU (Scale bar: 20 μm) **C** were performed to evaluate the effect of HDAC8 knockout and the K62 acetylation deficiency on the proliferation ability of HepG2 cells. Data shown are mean ± SD (*n* = 3) (*P < 0.05, **P < 0.01, ***P < 0.001). **D** Image of xenograft tumors resected from tumor-bearing NCG mice (*n* = 4). **E** Representative ^18^F-FDG micro-PET/CT images of two living NCG mice were conducted 5 weeks after subcutaneous inoculation. Images showed the obvious different ability of FDG uptake in different groups of xenografts. **F** The tumor tissues were examined by hematoxylin and eosin(H/E), anti-ac-lys-62-PKM2 antibodies, HDAC8, Ki-67, Cyclin D1 and Glut1(scale bar, 100 μm). The analysis of the intensity of cyclin D1 expression by image J. (*P < 0.05, one way ANOVA in **A**, **B**, **D**, **E**, **F**).

**Fig. 7:**
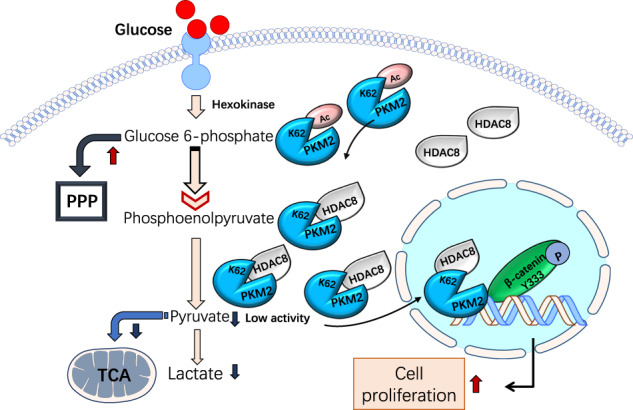
Schematic deacetylation of PKM2 at K62 in HCC. HDAC8 plays a crucial role in deacetylation of PKM2 at K62. Then the metabolic activity and nuclear relocation of PKM2 was narrowed down and increased separately.

Multiple studies have shown that HDAC8 promotes the growth and development of HCC. Consistently, HDAC8 inhibitors PCI-34051 and agonist TM-2-51 inhibited and promoted the growth of liver cancer cells, respectively (Fig. S5A). And, HDAC8 knockout also can significantly inhibit the growth of HCC cells, and the clone formation, and EdU incorporation further demonstrated the regulatory effect of HDAC8 on cell growth (Fig. S5B). Next, we analyzed the relationship between HDAC8-mediated PKM2-K62 deacetylation and HCC cell growth. Compared with the PKM2 wild-type or K62 site acetylation-deficient mutant, the K62 acetylation-deficient mutant significantly promoted the growth, clonal formation and EdU incorporation of HCC cells (Fig. 6A–C). Consistent with in vitro experiments, in vivo experiments have shown that knocking out HDAC8 can significantly inhibit the growth of tumor cells, while PKM2-K62R mutants instead of PKM2 wild-type can restore tumor cell growth defects caused by HDAC8 deficiency partly ((Fig. 6D). In addition, ^18^F-FDG-PET imaging showed that K62 acetylation-deficient HCC cells displayed glucose avidity phenotype (Fig. 6E). Protein level analysis by immunohistochemistry of confirmed the effect of the K62 acetylation level on promoting proliferation. As shown in Fig. 6F, HDAC8-KO group had the lowest cyclin D1 and GLUT1 protein expression. Just as we expected, HDAC8-KO/PKM2-K62R had a good performance. In conclusion, deacetylation at the K62 site of PKM2 protein plays an important role in regulating the growth of tumor cells (Fig. 7).

## Disscusion

PKM2 plays a key role in glucose metabolism and cell proliferation by changing enzyme activity and regulating gene transcription. A large amount of evidence indicates that post-translational modification is an important mechanism for PKM2 function and regulation of tumorigenesis^15,33^. Here, we found that the deacetylation of K62 is significantly related to the prognosis of liver cancer patients, and also to hepatocirrhosis. In addition, we demonstrated HDAC8 as an upstream deacetylase that regulates PKM2 K62 acetylation levels. Consistently, it has been reported that the HDAC inhibitor suberoylanilide hydroxamic acid can alleviate hepatocirrhosis by inhibiting the TGF-β1 signaling pathway^34^. Due to HDAC8 activation, K62 acetylation could be masked by the overall acetylation pattern, or could depend on cell type^35^. In addition, K62 and K305 may play different roles under various nutritional stresses or in different cell types, which also highlights the complex post-translational regulation of PKM2 protein. Especially in the case of HDAC8 activation, deacetylation at position K62 is particularly important for the regulation of PKM2 function. The phenotype of PKM2 K62 deacetylation is consistent with that of HDAC8 overexpression. This indicates that in the case of HDAC8 activation, deacetylation at K62 residue is particularly important for regulating PKM2 function^3,36,37^.

HDAC8 is localized in the cytoplasm and nucleus. In addition to the deacetylation of histone variants (e.g., H2A/H2B, H3, and H4), many nuclear targets of HDAC8 have been identified (e.g., structural maintenance of chromosome 3 [SMC3])^32,37^. Similarly, the glycolytic enzyme PGAM1 that catalyzes the conversion of 3-phosphoglycerate to 2-phosphoglycerate in the glycolysis pathway is also a regulatory target of HDAC8^38^. Recent evidence indicate that HDAC8 is phosphorylated by AMPK in lung cancer cells involved in glucose metal metabolism under hypoglycemia induction, and remains in the cytoplasm, resulting in upregulated expression of PGM1^39^. Here, we found that HDAC8 can directly bind and deacetylate to regulate PKM2 protein at K62 residue, which further deepens our understanding of HDAC8 in regulating glucose metabolism and driving tumorigenesis.

Cancer cells display a robust increase in glucose uptake, and there is a higher demand for metabolic intermediates biosynthesized to support rapid cell growth. PKM2 is a critical regulatory factor in directing glucose metabolism and the regulation of pyruvate kinase activity in proliferating cells may be particularly important to coordinate glucose metabolism with the synthesis of deoxynucleotides for DNA replication^15^. Acutely inhibiting the activity of PKM2 in proliferating cells leads to the temporary accumulation of upstream glycolysis intermediates and reduces oxygen consumption, making glucose metabolites available for anabolic processes^40^. The regulation of K62 acetylation level by HDAC8 affects the enzyme activity and protein stability of PKM2. As a result, pyruvate can be converted to lactic acid or oxidized to acetyl-CoA into the tricarboxylic acid cycle. The low activity of PKM2 can keep the levels of ATP and GTP low, thereby continuously activating phosphofructokinase-1, so the rate of glycolysis remains high. Therefore, inhibition of PKM2 activity can reprogram glucose metabolism, providing a large number of metabolic intermediates for biosynthesis, such as dihydroxyacetone phosphate for membrane lipid synthesis^17,27,41^.

In addition, PKM2 plays a carcinogenic role as a nuclear protein kinase^8,42,43^. Upon EGFR activation, acetylation of PKM2 Lys433 residue promotes its nuclear localization and upregulates the expression of cyclin D1^8^. K62 acetylation has a similar working model. We found that PKM2 K62 residues were deacetylated by HDAC8, causing PKM2 to translocate to nucleus and synergize with β-catenin transcription to upregulate the expression of cyclin D1. Our results confirmed that the regulation of PKM2 entry into the nucleus by HDAC8 is essential for the growth of tumor cells. In summary, we found that PKM2 is deacetylated by HDAC8 at K62 residue, which affects its role in glucose metabolism and gene transcription. The deacetylation status of K62 indicates a poor prognosis in HCC patients. The HDAC8-PKM2 pathway might provide new therapeutic targets for the treatment of HCC.

## Supplementary information

supplementary figure legends

supplementary figure s1

supplementary figure s2

supplementary figure s3

supplementary figure s4

supplementary figure s5
