# Supplementary material for: HDAC8-dependent deacetylation of PKM2 directs nuclear localization and glycolysis to promote proliferation in hepatocellular carcinoma
Source: Cell Death Dis. 2020 Dec 5;11(12):1036. doi: 10.1038/s41419-020-03212-3 (PMC7719180; doi:10.1038/s41419-020-03212-3)
Supplement: Supplementary file 1 — supplementary figure legends [file 41419_2020_3212_MOESM1_ESM.docx]

**Supplementary Figure Legends**

**Figure S1**: **A** the statistical analysis of t expression profiling of PKM in Hepatocellular carcinoma (normal vs. cancer) by GEPI2 website(<http://gepia2.cancer-pku.cn/#index>). **B** the statistical analysis of overall survival in liver cancer by GEPI2 website(<http://gepia2.cancer-pku.cn/#index>). **C** Query datasets for GSE4465 which is about HDACI treatment (TSA,0.5uM for 24hrs) of hepG2 cells (control vs. TSA) from website <https://www.ncbi.nlm.nih.gov/geo/>, and these datasets were analyzed by GEO2R. Then the data for statistical analysis was mapped by R project ,log FC＞0.7, P＜0.05. **D**, **E** Date of acetylation sites of PKM2 was from <https://www.phosphosite.org/homeAction.action>. **F** The K62ac Specific antibody validation. **G** The validation of specificity of custom-designed acetylated PKM2 K62 antibody

**Figure S2 A** The validation of HDAC8-KO cells. **B** The possibility of HDAC8 acting on PKM2 was predicted through different web sites (<http://cssn.biocuckoo.org>).

**Figure S3 EGF stimulation on cells, the acetylation of K62 PKM2 was lowed.**

HepG2 cells were planted in 6-well plate, cultured overnight. Then cells were transfected with Flag- PKM2 plasmid for 48 h, during a specified time, EGF(100 ng/ml) was added. After immunoprecipitated with Flag affinity gel, acetylation of K62 was determined by western blotting.

**Figure S4 HDAC8 promotes nuclear translocation of PKM2.** HepG2 cells were transfected with siHDAC8 (or negative control siRNA) (**a**) or HA-HDAC8 plasmids (or vector plasmid) (**b**), after 24h, cells were placed on glass coverslips, cultured for another 24h. Anti-PKM2 (red) and anti-HDAC8 (green)（bsm-52088R, Bioss） were used for immunofluorescence (scale bar: 50 μm). The line profiles of the mean fluorescence intensity of PKM2 and DAPI signals were measured by ImageJ software. (Scale bars=20 μm.)

**Figure S5 HDAC8 promotes HCC cell lines proliferation. a** Inhibition or activation of HDAC8 significantly inhibited or promoted cell proliferation in HepG2. **b** HDCA8 KO could impede cells proliferation. **c** In SMMC-7721, the R mutation of PKM2 at K62, promotes cell proliferation (**p*＜0.05，***p*＜0.01). SMMC-7721 cells stably expressing PKM2-WT, K62R or K62Q were injected subcutaneously into the right flank of nude mice(n=6). **d** ^18^F-FDG micro-PET/CT images were conducted for nude mice, 3 weeks after injection before sacrificed. The white triangle shows the tumor. SUVmax were measured for the ability of ^18^F –FDG(n=4). **e** After three weeks, tumors were isolated from those mice, the volumes were recorded. P < 0.05 was considered significant. **f** The tumor tissues were examined by hematoxylin and eosin(H/E), anti-ac-lys-62-PKM2 antibodies, Ki-67 and Cyclin D1 (scale bar, 50 μm). The analysis of the intensity of cyclin D1 expression by image J. (Student’s t-test in a, one way ANOVA in d, g, h, j).
