# Supplementary figures and images for: HDAC8-dependent deacetylation of PKM2 directs nuclear localization and glycolysis to promote proliferation in hepatocellular carcinoma
Source: Cell Death Dis. 2020 Dec 5;11(12):1036. doi: 10.1038/s41419-020-03212-3 (PMC7719180; doi:10.1038/s41419-020-03212-3)

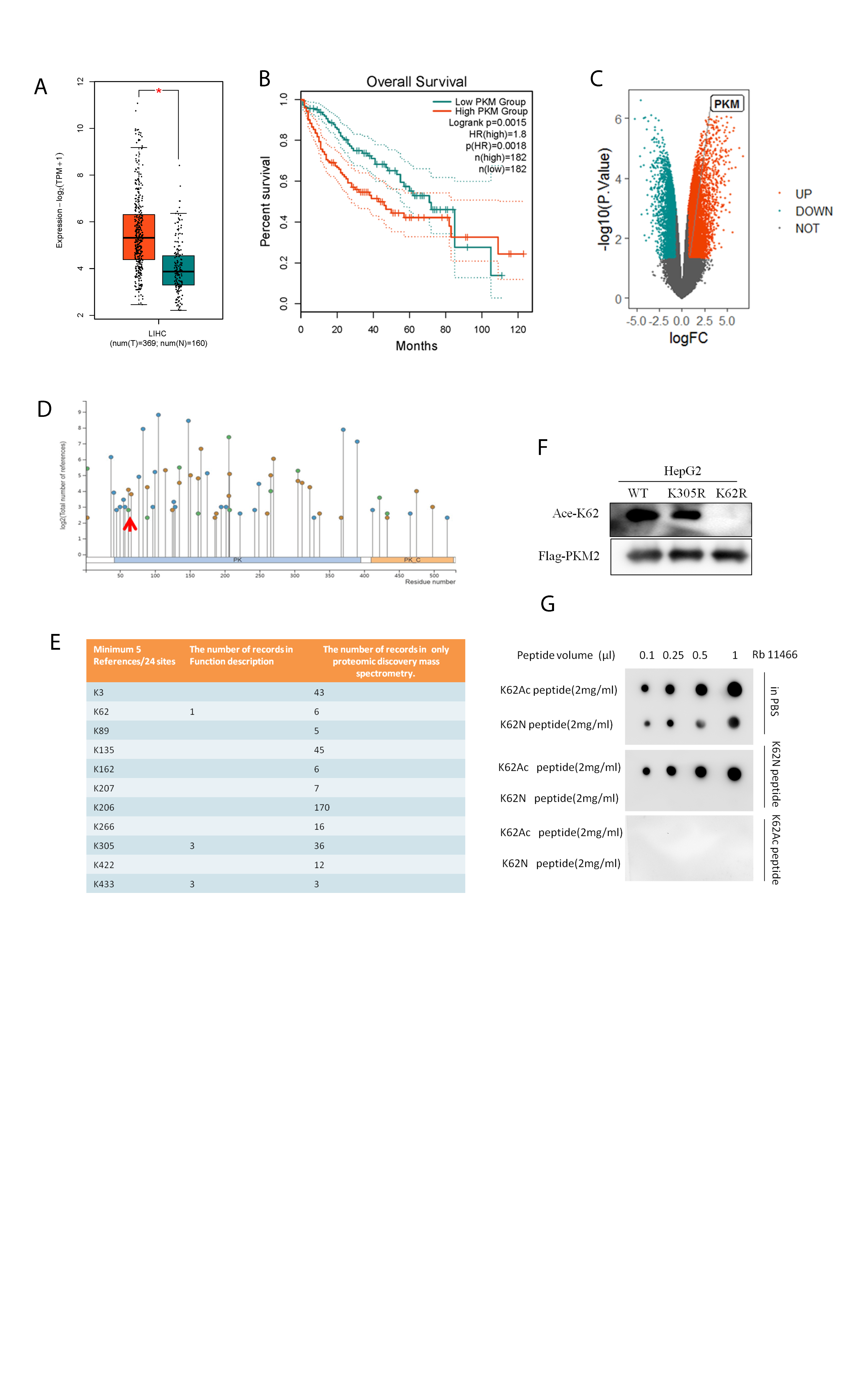

Supplement: Supplementary file 2 — supplementary figure s1 [file 41419_2020_3212_MOESM2_ESM.tif]

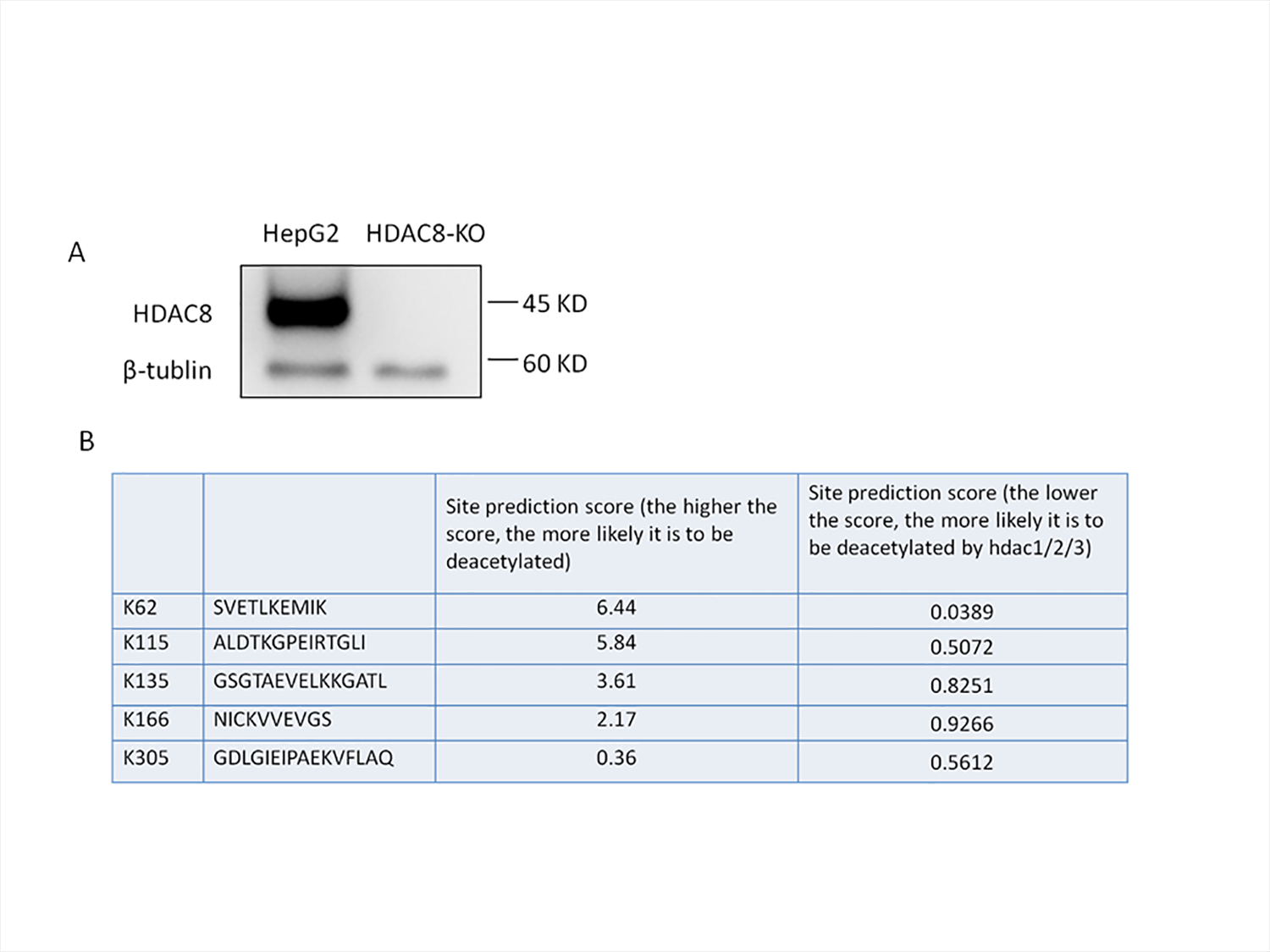

Supplement: Supplementary file 3 — supplementary figure s2 [file 41419_2020_3212_MOESM3_ESM.tif]

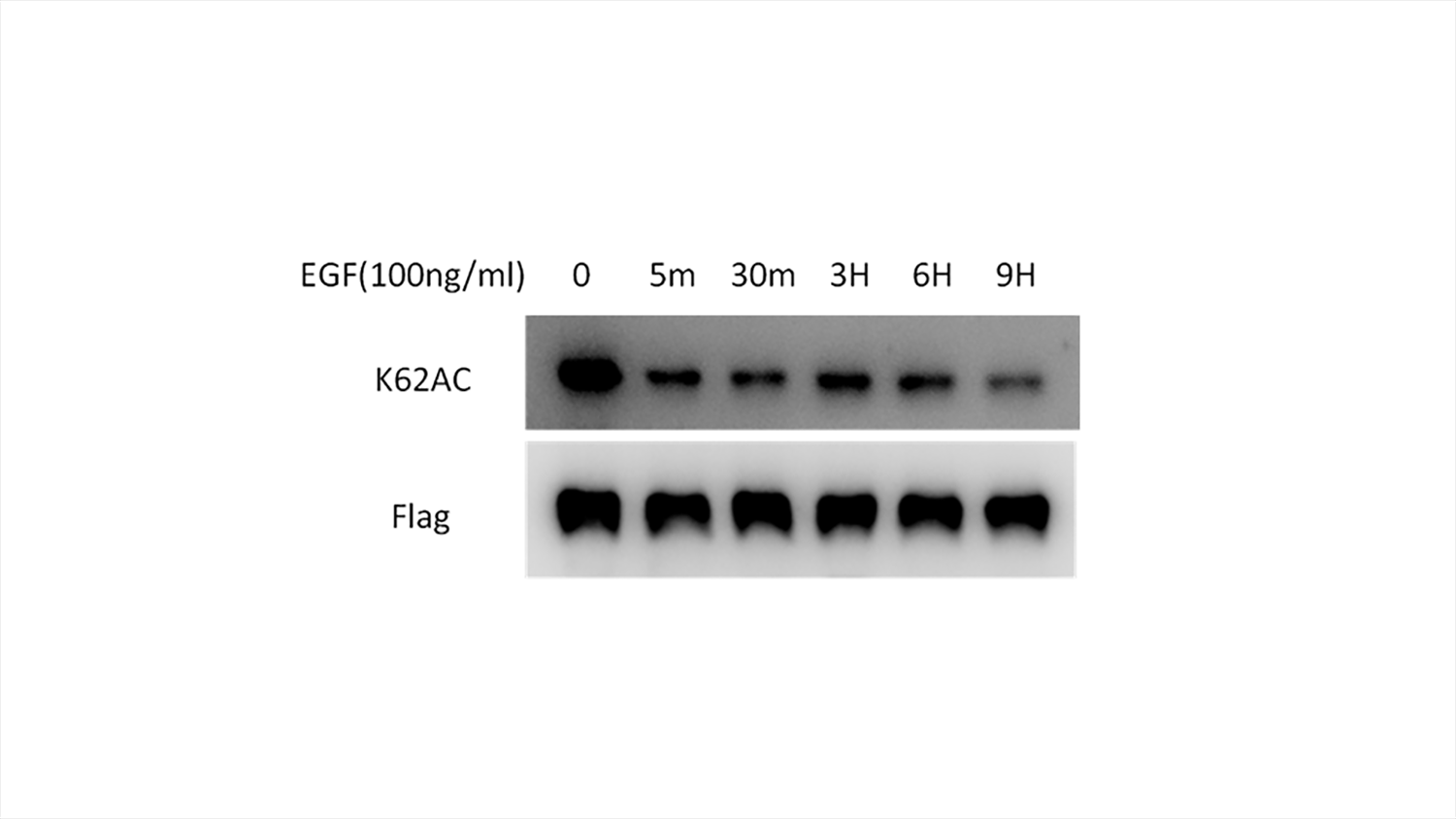

Supplement: Supplementary file 4 — supplementary figure s3 [file 41419_2020_3212_MOESM4_ESM.tif]

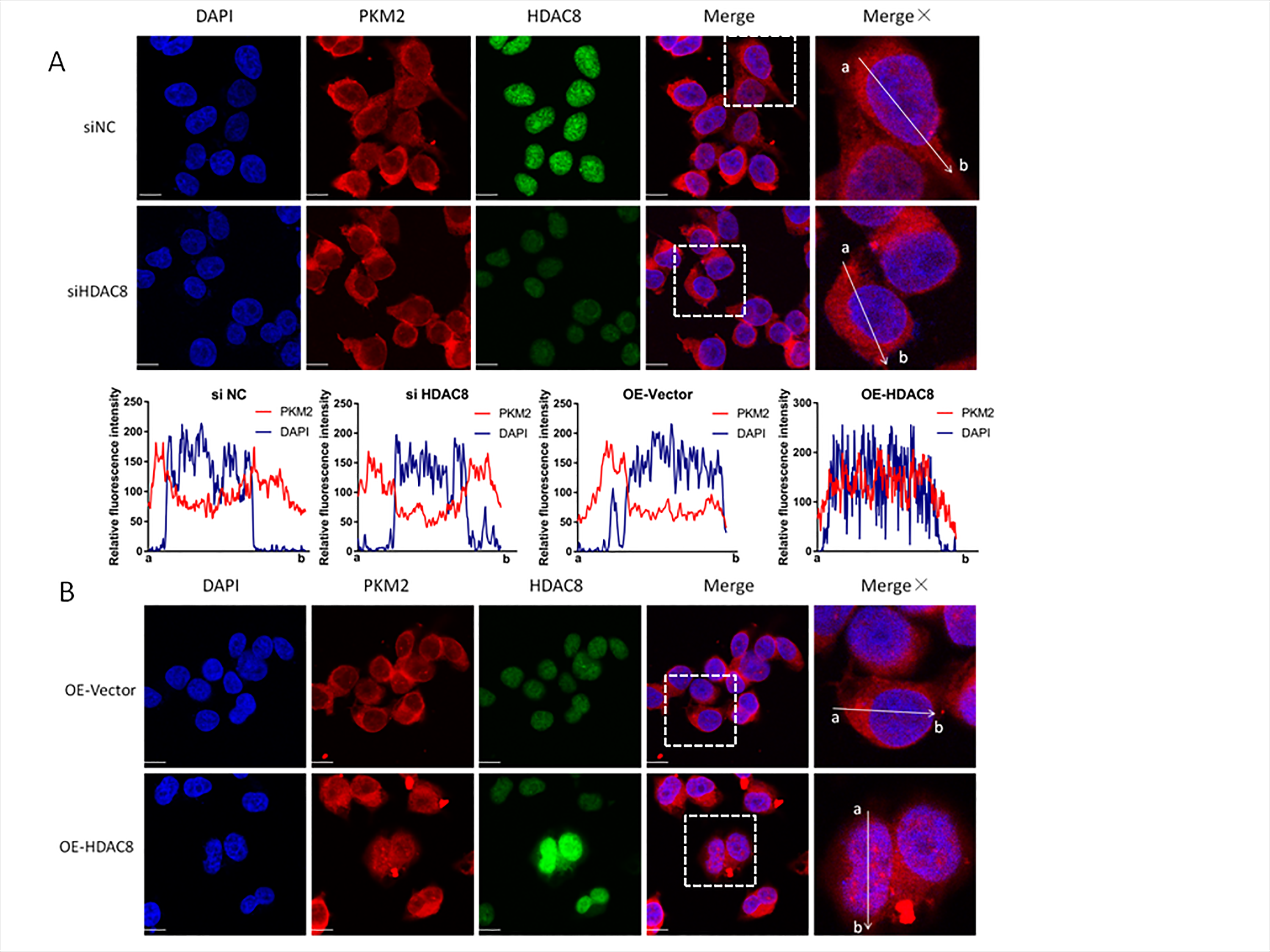

Supplement: Supplementary file 5 — supplementary figure s4 [file 41419_2020_3212_MOESM5_ESM.tif]

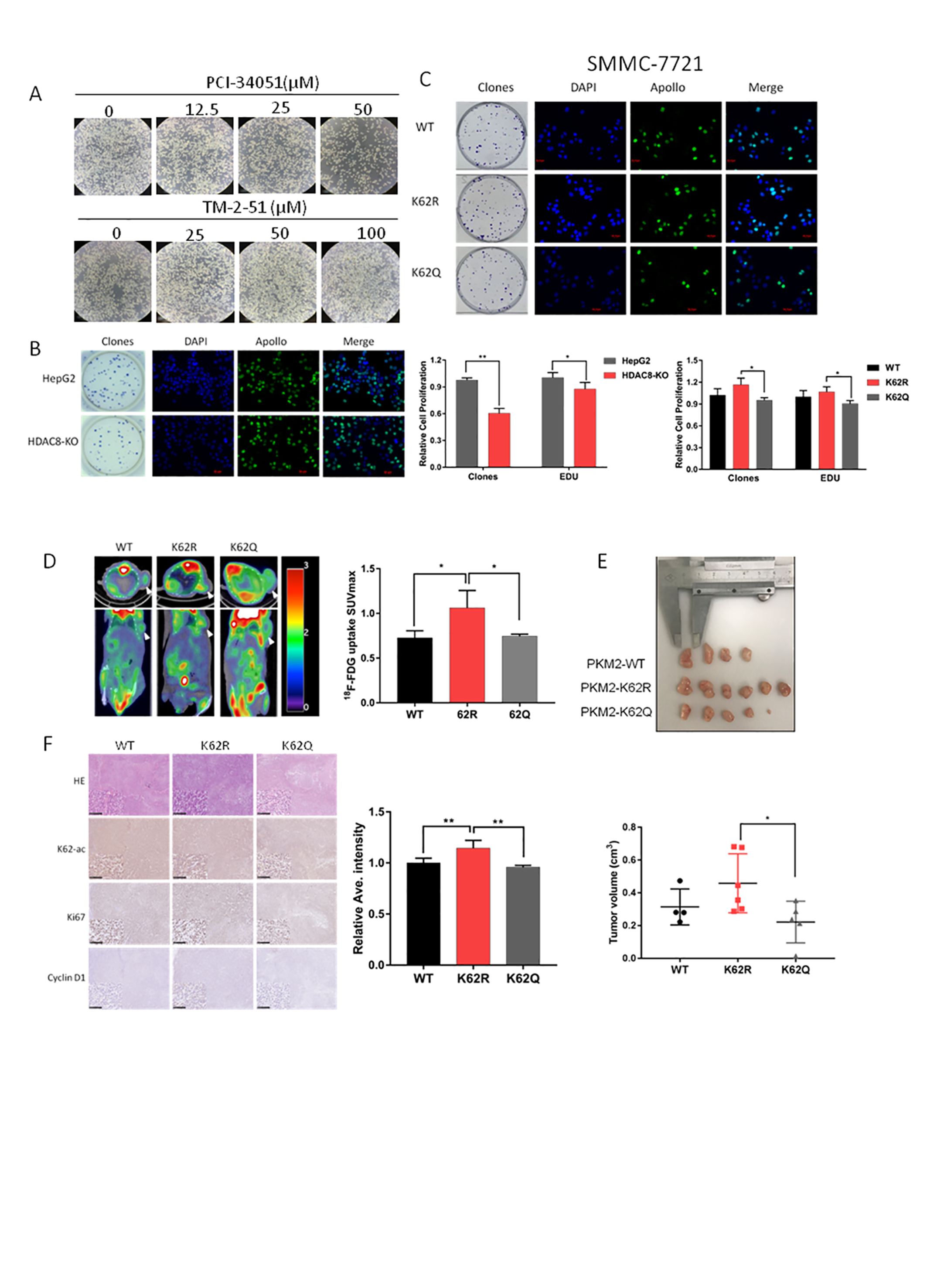

Supplement: Supplementary file 6 — supplementary figure s5 [file 41419_2020_3212_MOESM6_ESM.tif]
